# Species-specific gene regulatory network rewiring mediated by the GATA-type regulator NsdD in *Aspergillus*

**DOI:** 10.1128/mbio.01181-25

**Published:** 2025-07-03

**Authors:** Heungyun Moon, Mi-Kyung Lee, Junha Shin, Sung Chul Park, Julio C. Rivera Vazquez, Daniel Amador-Noguez, Nancy P. Keller, Hee-Soo Park, Kap-Hoon Han, Jae-Hyuk Yu

**Affiliations:** 1 Department of Plant Pathology, University of Wisconsin-Madison 312673, Madison, Wisconsin, USA; 2 Department of Bacteriology, University of Wisconsin-Madison 205263, Madison, Wisconsin, USA; 3 Great Lakes Bioenergy Research Center, University of Wisconsin-Madison 5228, Madison, Wisconsin, USA; 4 Biological Resource Center/Korean Collection for Type Cultures, Korea Research Institute of Bioscience and Biotechnology 54679, Jeongeup, Republic of Korea; 5 Wisconsin Institute for Discovery, University of Wisconsin-Madison 827307 , Madison, Wisconsin, USA; 6 Department of Medical Microbiology and Immunology, University of Wisconsin-Madison 732057, Madison, Wisconsin, USA; 7 School of Food Science and Biotechnology, Kyungpook National University 34986, Daegu, Republic of Korea; 8 Department of Pharmaceutical Engineering, Woosuk University 35031, Wanju, Republic of Korea; Nanjing Agricultural University, Nanjing, Jiangsu, China

**Keywords:** *Aspergillus*, GATA transcription factor, NsdD, development, metabolism, multiomics, gene regulatory network, network rewiring

## Abstract

The evolutionarily conserved GATA-type transcription factor (TF) NsdD regulates sexual and asexual development as well as secondary metabolism in various *Aspergillus* species. Despite its well-known multifunctionality, the mechanisms by which NsdD coordinates such diverse biological processes remain unclear. To address this gap, we have conducted network-based multiomics analyses in two distantly related species, *Aspergillus nidulans* and *Aspergillus flavus*. Transcriptomic profiling reveals that NsdD regulates gene expression in a cell type- and species-specific manner. The potential evolutionary conservation of NsdD was tested by a cross-complementation experiment in which the *A. nidulans nsdD* gene was introduced into the *A. flavus* Δ*nsdD* mutant. This partially restored key phenotypes and gene expression profiles but failed to fully recapitulate wild-type regulation, suggesting species-specific functionality. To further dissect NsdD’s roles, we have performed genome-wide ChIP-seq analyses and identified 502 and 674 potential direct targets in *A. nidulans* and *A. flavus*, respectively, including major developmental and metabolic regulators such as *veA*, *flbD*, *brlA*, *vosA*, *rosA*, and *laeA*. Motif analysis reveals a conserved NsdD binding site (5′-GATCT-3′), designated as the NsdD response element. Network analyses uncover core regulatory modules and reveal extensive gene regulatory network (GRN) rewiring between the two species. While NsdD governs conserved biological processes, divergence in its direct targets and downstream interactions contributes to species-specific traits, including differences in asexual morphology and production of sterigmatocystin/aflatoxin. This study provides the first genome-wide comparative map of NsdD-mediated GRNs in filamentous fungi and highlights how evolutionary rewiring allows a conserved TF to acquire distinct regulatory functions across species.

**IMPORTANCE:**

Multifunctional TFs are central in coordinating development and metabolism in filamentous fungi. In this study, we systematically dissect the regulatory functions of NsdD, a highly conserved GATA-type TF in Pezizomycotina, using network-based multi-omics approaches in two distantly related species, *A. nidulans* and *A. flavus*. Our analyses reveal that NsdD governs fungal development and metabolism through species-specific GRNs, directly targeting key upstream regulators and genes involved in core cellular processes. These regulatory distinctions underlie the morphological and metabolic differences observed between the two species. Notably, our cross-species comparison uncovers extensive GRN rewiring, demonstrating how evolutionary divergence can reshape transcriptional networks even under conserved regulatory control. The resulting GRN maps offer a valuable framework for understanding gene regulation in *Aspergillus* and provide a foundation for broader studies on the evolution of transcriptional networks and conserved regulatory factors in filamentous fungi.

## INTRODUCTION

Fungi profoundly impact human life due to their medical, environmental, agricultural, and industrial importance. They act as pathogens and antibiotic producers, contribute to nutrient cycling, play roles in food fermentation and spoilage, and serve as key organisms in agricultural and biotechnological applications. Among filamentous fungi, species in the phylum Ascomycota primarily reproduce by forming asexual spores (conidia), which are readily dispersed by wind, water, or physical disturbance. This mode of reproduction enhances their ability to survive, spread, and colonize new environments (1, 2). Notably, in certain fungi, sexual and asexual development is closely intertwined with secondary metabolism. Multiple studies have shown that mutants impaired in sexual/asexual development often exhibit defects in the production of mycotoxins such as aflatoxins (AFs) and sterigmatocystin (ST) (3–5). Due to its prevalence across fungal species, asexual sporulation provides a powerful model for exploring genotype–phenotype relationships within species and examining gene regulatory network (GRN) rewiring across species.

*Aspergillus* is among the most diverse fungal genera, comprising over 340 recognized species (6). The biological diversity of *Aspergillus* spp. and the availability of the well-established model organism *A. nidulans* make this genus an ideal system for studying GRN rewiring and its influence on cellular and molecular phenotypes. Asexual development (conidiation) in *Aspergillus* is tightly regulated by the balanced activity of positive regulators (e.g., FluG and FLBs) and negative regulators (e.g., SfgA, NsdD, and VosA) (7–11). Activation of *brlA*, which encodes a C_2_H_2_ zinc finger transcription factor (TF), initiates the development of multicellular reproductive structures called conidiophores. This is followed by the sequential activation of *abaA* and *wetA*, which function at later stages to ensure proper spore maturation, resulting in chains of non-motile conidia (12–14). Importantly, proper asexual development requires the timely removal of the repressors NsdD and VosA, which must occur before the activation of *brlA* (15). Conidiophore morphology differs markedly between species. For example, the distantly related species, *A. nidulans* and *A. flavus*, possess more divergent genomes than humans and chickens, forming structurally distinct conidiophores (16). Markedly, some *A. flavus* conidiophores lack metulae, the structural elements that connect the vesicle to the phialide in *A. nidulans*. In addition to asexual reproduction, approximately 36% of *Aspergillus* spp. are also capable of sexual reproduction (17). During the sexual developmental stage, fungi produce ascospores within specialized fruiting bodies called cleistothecia. In *A. nidulans*, a homothallic (self-fertile) species, mycelia aggregate to form Hülle cells, which function as nuclear reservoirs and provide structural support for cleistothecia (18). By contrast, *A. flavus* is heterothallic, requiring a compatible mating partner for sexual reproduction. It forms sclerotia, hardened structures that function as dormant survival bodies and reproductive organs (19). Several sexual developmental regulators—such as NsdD, VeA, SteA, RosA, and CryA—have been identified, and their functions are closely intertwined with secondary metabolism, which underlies the production of antibiotic, antiviral, antitumor, immunosuppressive, phytotoxic, and mycotoxic compounds (3, 20, 21).

**Fig 1:**
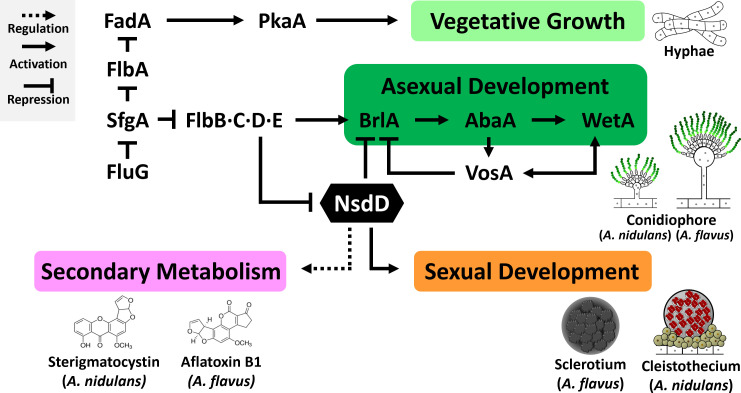
Schematic overview of NsdD functions in development and secondary metabolism. Solid arrows indicate positive (activating) regulation. Dotted arrows indicate unspecified regulation (either activating or repressing). Blunt-ended lines indicate repression. This model is adapted and modified from reference 22.

Among TFs, NsdD—a GATA-type regulator—plays a pivotal role in controlling fungal development and metabolism (Fig. 1) (22). Studies have shown that NsdD acts as a positive regulator of sexual development, as the *nsdD* null mutant fails to form fruiting bodies even under conditions favorable for sexual reproduction. Conversely, the overexpression of *nsdD* promotes fruiting body formation and confers tolerance to factors that normally inhibit sexual development in *Aspergillus* (23–25). On the other hand, NsdD is a key repressor of asexual development and negatively regulates *brlA* expression, thereby preventing premature initiation of conidiation (7, 15). NsdD directly binds to the *brlA* promoter and collaborates with VosA to repress *brlA* expression. The deletion of *nsdD* results in accelerated and premature conidiation, with mutants forming asexual structures even under submerged liquid culture conditions where conidiation is typically suppressed. In addition to regulating timing, NsdD also influences the morphology of asexual structures. In *A. flavus*, *nsdD* deletion leads to conidiophores that are approximately 10 times shorter, resembling those of wild-type (WT) *A. nidulans* (15, 25). NsdD also plays a crucial role in regulating mycotoxin biosynthesis, including ST and AFs. Interestingly, although ST is a direct precursor of AF, *nsdD* deletion enhances ST production in *A. nidulans* but abolishes AF production in *A. flavus*, indicating a species-specific regulatory role. NsdD is among the most evolutionarily conserved GATA-type TFs in Pezizomycotina—a subclass of Ascomycota that includes genera such as *Aspergillus*, *Penicillium*, *Sclerotinia*, *Coccidioides*, *Ajellomyces*, and *Fusarium* (26). Although the DNA-binding domain of NsdD is highly conserved—exhibiting approximately 95% sequence identity across *Aspergillus* spp.—the full-length protein shows considerable variation. For example, NsdD from *A. nidulans* and *A. flavus* shares only 75% overall amino acid similarity, highlighting its potential for evolutionary divergence despite the conservation of its DNA-binding domain (Fig. S1).

This study focuses on elucidating the species-specific regulatory roles of the conserved GATA-type TF NsdD in *A. nidulans* and *A. flavus*, with particular emphasis on its functions during vegetative growth and asexual development. While NsdD is known to influence both sexual and asexual processes in filamentous fungi, capturing its role in sexual development remains technically challenging due to the complexity and variability of the sexual cycle across species. By applying a multiomics approach—including transcriptomics, chromatin immunoprecipitation followed by sequencing (ChIP-seq), and metabolomics—our work provides the first genome-wide comparative analysis of NsdD-mediated GRNs in filamentous fungi. We reveal that although NsdD governs conserved developmental and metabolic processes, its regulatory output is rewired in a species-specific manner, leading to distinct morphological and biochemical phenotypes. These findings highlight the evolutionary plasticity of fungal GRNs and underscore the broader biological principle that conserved TFs can acquire divergent functions through network rewiring. Our study offers a valuable framework for dissecting transcriptional network evolution and may inform strategies for manipulating fungal development and metabolite production in both basic and applied contexts.

## RESULTS

### Species-specific and cell type-dependent gene regulation of NsdD

**Fig 2:**
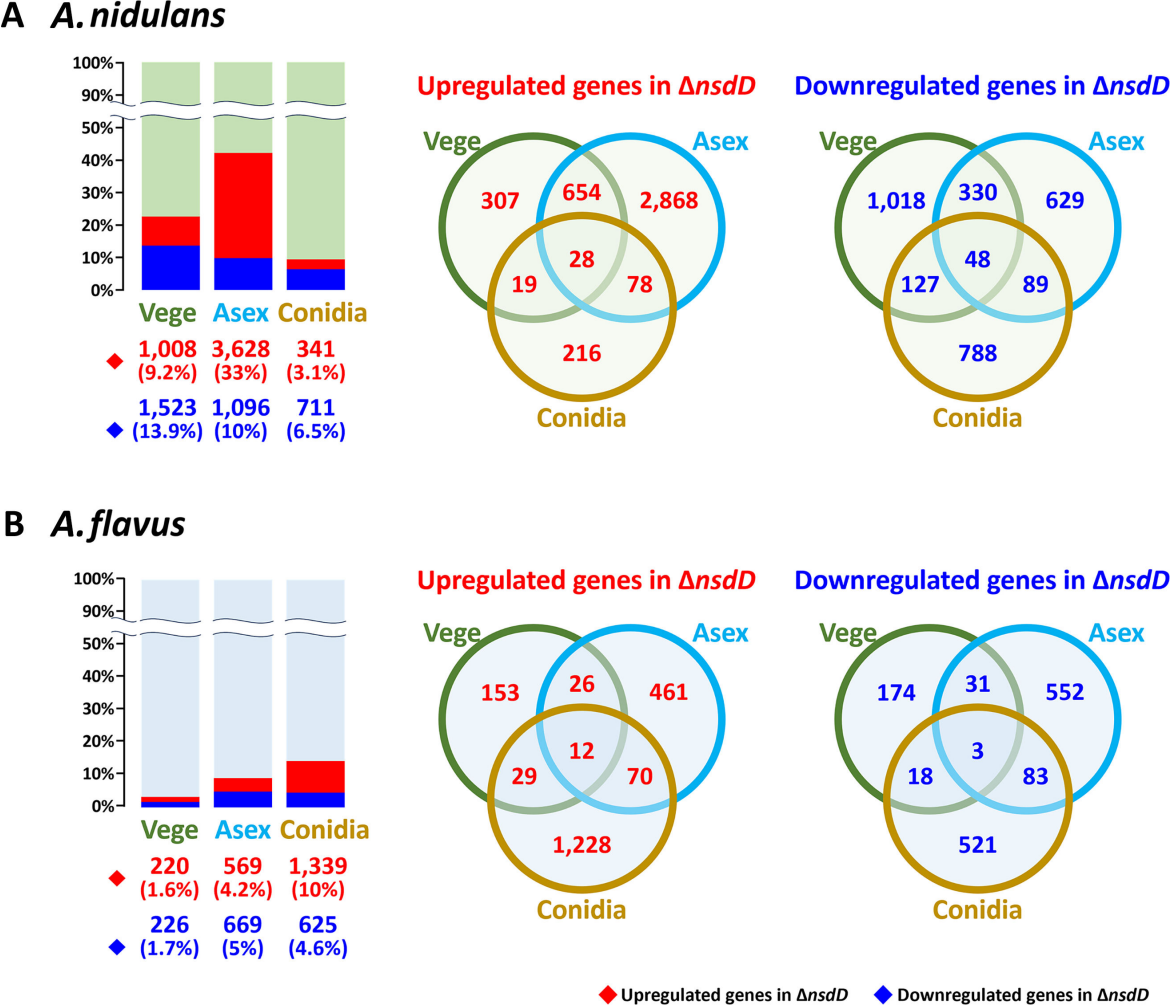
Summary of transcriptomic analyses in *A. nidulans* and *A. flavus*. Differential gene expression in *A. nidulans* (**A**) and *A. flavus* (**B**) Δ*nsdD* mutants compared to WT. RNA-seq was performed using three cell types: vegetatively grown cells (Vege, 36 h), asexually developing cells (Asex, 24 h), and conidia (2 days). Red and blue numbers indicate the number of upregulated and downregulated genes in Δ*nsdD*, respectively.

NsdD is a key TF regulating developmental processes and secondary metabolism in *Aspergillus* spp. However, its regulatory roles across different cell types and developmental stages remain unclear. To address this, we analyzed the transcriptome of three cell types: vegetative cells, asexually developing cells, and conidia. These analyses were performed in WT and Δ*nsdD* strains of *A. nidulans* and *A. flavus*. Our analyses have revealed that the regulatory effects of NsdD vary significantly, depending on species and cell type. In *A. nidulans*, 23.03%, 42.99%, and 9.57% of total genes were differentially expressed in Δ*nsdD* vegetative cells, asexual cells, and conidia, respectively. In contrast, in *A. flavus*, 3.3%, 9.18%, and 14.56% of genes showed differential expression (*P* < 0.05, |log_2_FC| > 1) (Fig. 2). Each cell type exhibited distinct gene expression profiles in Δ*nsdD*, suggesting that NsdD establishes a species-specific and cell type-dependent gene regulatory network.

**Fig 3:**
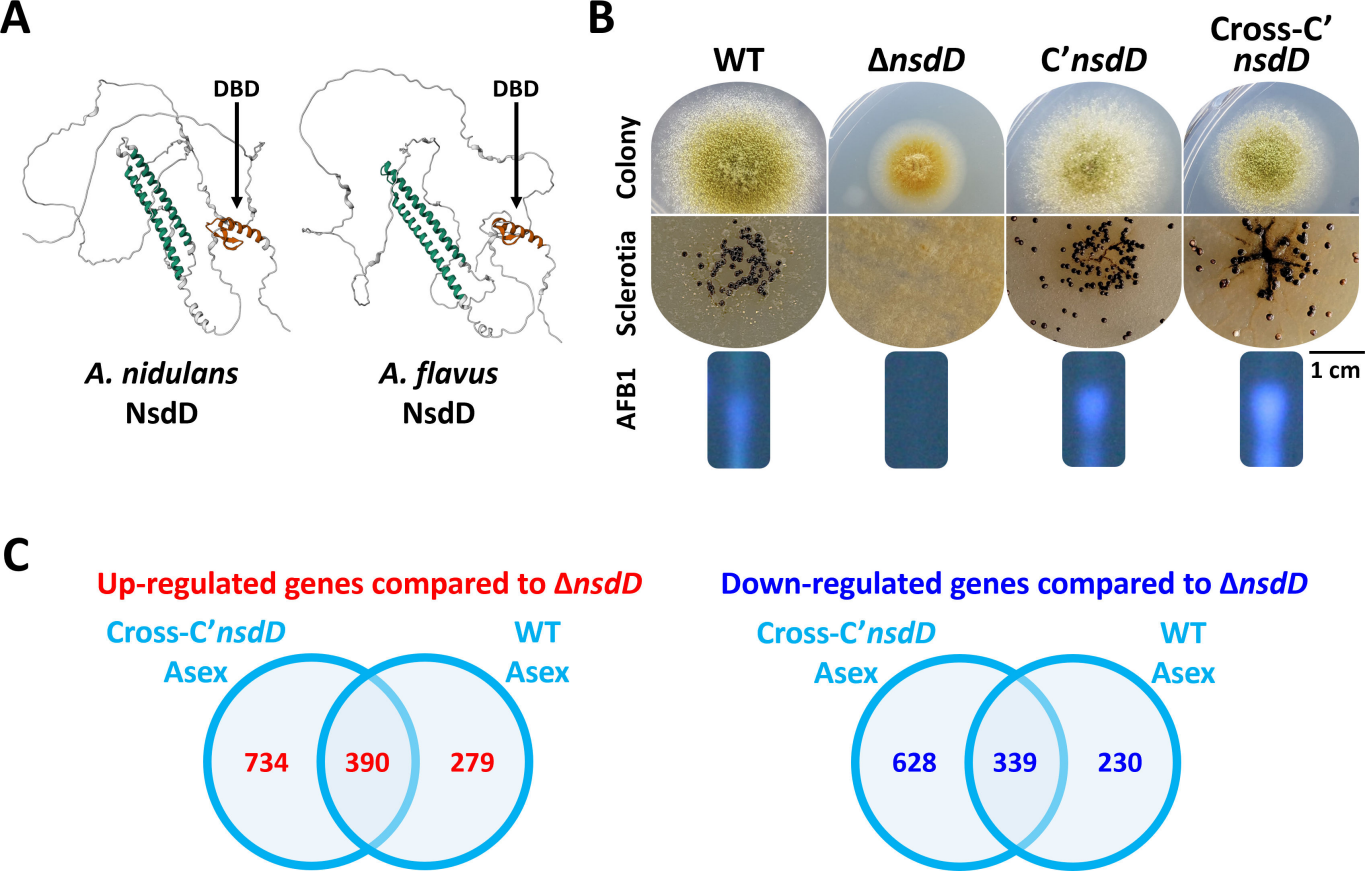
Cross-complementation analysis of NsdD between *A. nidulans* and *A. flavus*. (**A**) Predicted 3D structures of NsdD proteins from *A. nidulans* and *A. flavus* generated by AlphaFold (27). The two NsdD proteins share 75% overall amino acid similarity, with over 95% identity in their DNA-binding domains (DBDs). (**B**) Representative colony morphology of *A. flavus*, including sclerotia formation and AFB1 production. C′*nsdD*, *A. flavus* complemented strain expressing native *AflnsdD*; cross-C′*nsdD*, *A. flavus* cross-complemented strain expressing *AninsdD*. (**C**) Transcriptomic comparison of cross-C′*nsdD* and WT strains in Asex. Expression levels are shown relative to the Δ*nsdD* mutant.

To further investigate NsdD’s potential evolutionary conservancy and species-specific regulatory functions, we expressed *A. nidulans nsdD* in *A. flavus* Δ*nsdD* mutant strains and performed phenotypic and transcriptomic analyses during the asexual development stage (Fig. 3). Cross-complemented strains exhibited partial recovery of key phenotypic characteristics, including sclerotia formation and aflatoxin production (Fig. 3B) and gene expression patterns, yet failed to fully reconstitute the transcriptomic responses compared to what was observed in WT (Fig. 3C). In cross-complemented strains, 734 genes were upregulated compared to the *A. flavus* Δ*nsdD* mutant, of which 390 overlapped with the WT expression profile and 279 were specific to WT. Similarly, 628 genes were downregulated, with 339 overlapping with WT and 230 being WT-specific. These results suggest that although NsdD retains core regulatory functions across species, its downstream gene regulatory network is largely species-specific and likely shaped by additional regulatory factors unique to each genetic background.

To further explore NsdD’s regulatory roles, we performed functional enrichment analyses using Gene Ontology (GO) terms across three developmental stages in *A. nidulans* and *A. flavus*. In *A. nidulans*, NsdD activated secondary and carbohydrate metabolism while repressing protein metabolic processes during vegetative growth and asexual development. In conidia, it promoted cell wall metabolism and secondary metabolism while inhibiting carbohydrate transport and amino acid catabolism (Fig. S2). Similarly, in *A. flavus*, NsdD was essential for secondary metabolism, including mycotoxin production, while repressing carbohydrate and protein metabolism during vegetative and asexual stages. In conidia, NsdD suppressed transcription while promoting amino acid catabolism (Fig. S3). Overall, these findings indicate that NsdD dynamically regulates transcription, metabolism, and development across different stages in *Aspergillus* spp., reinforcing its role in species-specific and cell type-dependent gene regulatory networks.

### Primary metabolomics in the Δ*nsdD* conidia

**Fig 4:**
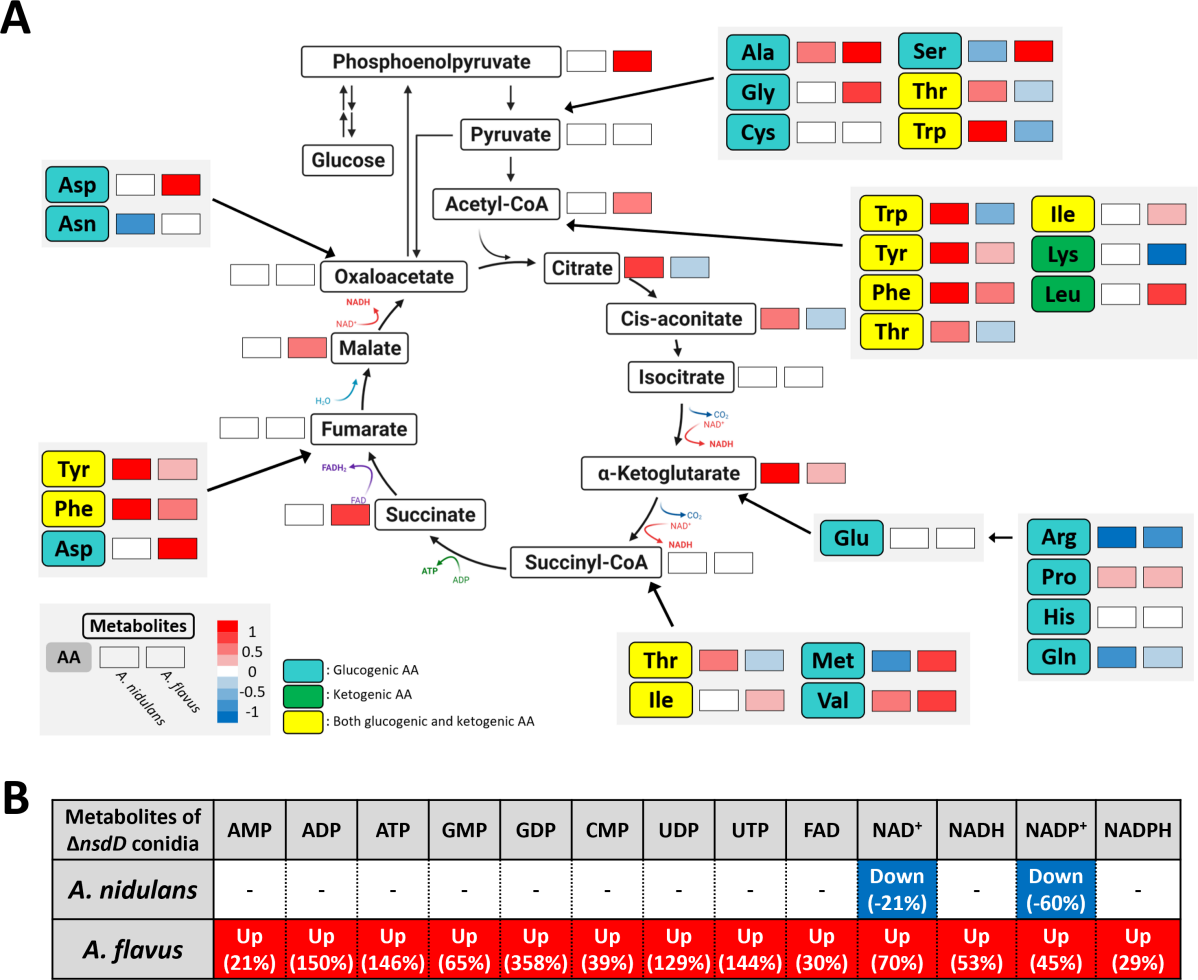
Alteration of conidial accumulation of primary metabolite by Δ*nsdD*. (**A**) Schematic representation of NsdD-mediated regulation of primary metabolism. Metabolites involved in glycolysis, the citric acid cycle, and amino acid (AA) biosynthesis are shown. Paired rectangles indicate changes in metabolite abundance in *A. nidulans* (left) and *A. flavus* (right) conidia. Red and blue indicate increased and decreased metabolite levels, respectively, in the Δ*nsdD* mutant. Glucogenic AAs are catabolized to pyruvate or other glucose precursors, whereas ketogenic AAs are catabolized to acetyl-CoA. (**B**) Relative abundances of energy-related metabolites, including ATP, NADH, and NADPH in the Δ*nsdD* conidia of *A. nidulans* and *A. flavus*.

Functional analyses have shown that *nsdD* deletion affects the expression of genes related to primary metabolism, including amino acid metabolism. To determine whether these led to the altered primary metabolite production, we quantified key metabolites, including amino acids, citric acid cycle intermediates, and energy-related molecules such as ATP, NAD, and NADP, in WT and the *nsdD* mutant conidia of *A. nidulans* and *A. flavus* (Fig. 4). The *nsdD* deletion altered primary metabolite profiles in both species (Table S1). In the *A. nidulans* Δ*nsdD* conidia, 111 metabolites were significantly affected (*P* < 0.05, |log_2_FC| > 1), with a nearly equal split between increased (52) and decreased (59) accumulations of metabolites. In contrast, the *A. flavus* Δ*nsdD* conidia showed a greater shift, with 167 altered metabolites, most of which showed increased (97) rather than decreased (70) accumulation. Twelve amino acids, including alanine, arginine, and methionine, along with three citric acid cycle intermediates (citrate, aconitate, and α-ketoglutarate), were altered in the *A. nidulans* Δ*nsdD* conidia. In contrast, the *A. flavus* Δ*nsdD* conidia exhibited changes in 16 out of 20 amino acids and six citric acid cycle metabolites, including acetyl CoA, citrate, and succinate (Fig. 4A). Notably, energy metabolism-related metabolites were significantly elevated in the *A. flavus* Δ*nsdD* conidia, showing up to a 358 percent increase compared to WT. In contrast, the *A. nidulans* Δ*nsdD* conidia showed significant changes only in NAD^+^ and NADP^+^ levels (Fig. 4B). These findings indicate that NsdD modulates primary metabolism in a species-specific manner, with a particularly strong influence on energy metabolism in *A. flavus*.

### Regulatory roles of NsdD in secondary metabolism

Previous studies have shown that NsdD represses sterigmatocystin production in *A. nidulans* but activates aflatoxin biosynthesis in *A. flavus* (25, 28). Consistent with this, GO term analyses revealed that many differentially expressed genes (DEGs) in Δ*nsdD* were involved in secondary metabolism, including mycotoxin biosynthesis (Fig. S2 and S3). To further investigate NsdD’s role in regulating secondary metabolites, we analyzed the expression of biosynthetic gene clusters (BGCs) associated with sterigmatocystin, aflatoxin, asperfuranone, aspernidine, asterriquinone (ARQ), austinol, dehydroaustinol, imizoquin, monodictyphenone, penicillin, and ustiloxin.

To assess the impact of NsdD on secondary metabolism, we performed ultra-high-performance liquid chromatography coupled with high-resolution mass spectrometry (UHPLC-HRMS)-based metabolomic analyses in *A. nidulans* and *A. flavus*. A total of 555 and 195 secondary metabolites were significantly altered (*P* < 0.05, |log_2_FC| > 1) in *A. nidulans* and *A. flavus* Δ*nsdD*, respectively (Table S2). In *A. nidulans* Δ*nsdD*, 381 metabolites showed decreased production, while 174 increased. Notably, 196 metabolites detected in WT were absent in Δ*nsdD*, whereas nine metabolites were exclusively found in Δ*nsdD*. In *A. flavus* Δ*nsdD*, 88 metabolites were downregulated and 107 were upregulated. Additionally, 26 metabolites detected in WT were absent in Δ*nsdD*, while 21 were uniquely present in Δ*nsdD*. These results indicate that NsdD plays a central role in secondary metabolism in both species.

**Fig 5:**
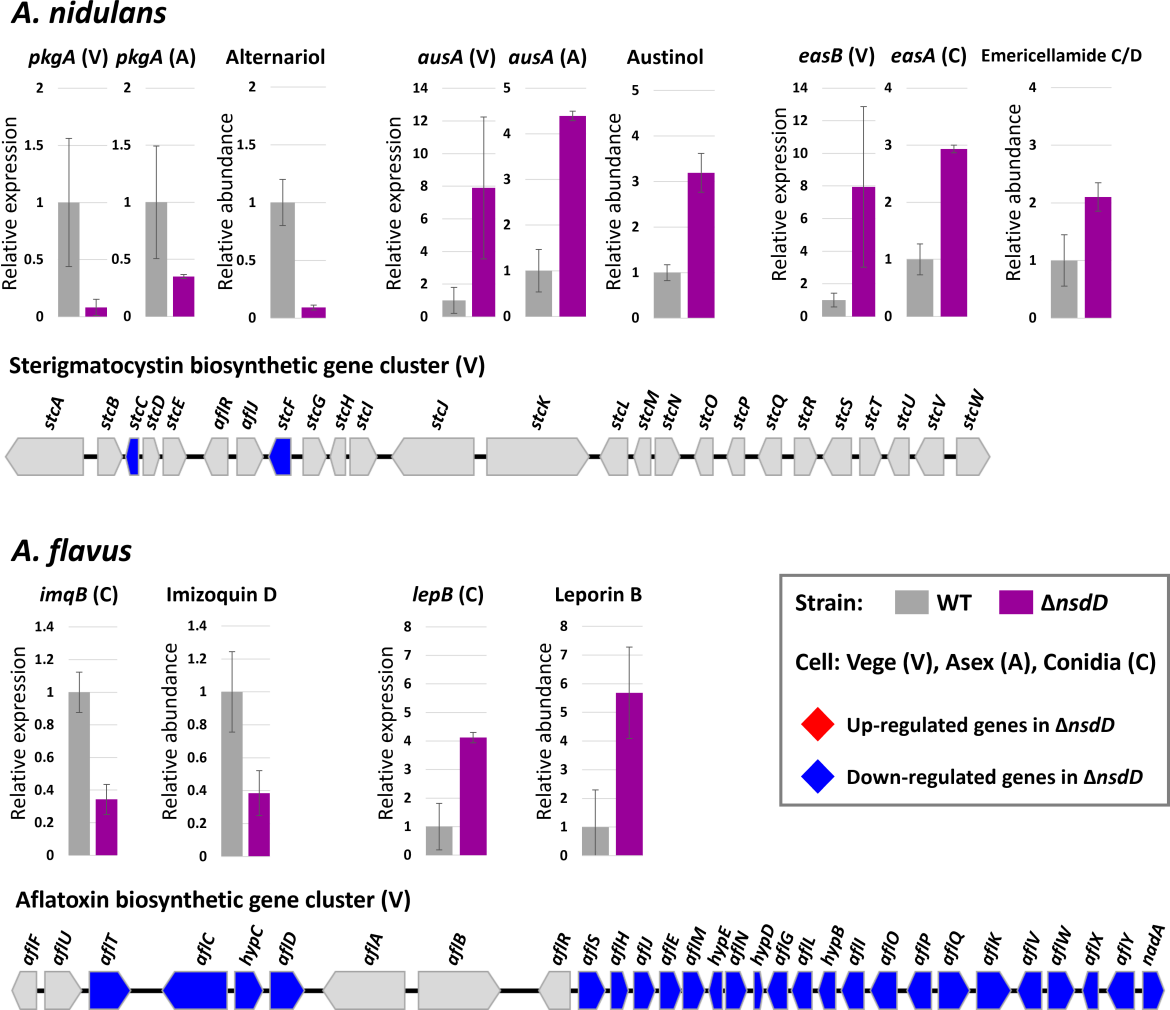
Regulatory roles of NsdD in the production of known secondary metabolites. Bar graphs show the relative expression levels of biosynthetic backbone genes (left) and the corresponding metabolite abundances (right) in WT and Δ*nsdD* strains of *A. nidulans* and *A. flavus*. Gene names and metabolite names are indicated above each panel. Sample types used for transcriptomic data are indicated as follows: V, vegetative cells; A, asexually developing cells; C, conidia. All differences shown are statistically significant (*P* < 0.05). The structures of the ST and AF BGCs are shown below, with gene expression patterns in Δ*nsdD*.

Next, we identified known secondary metabolites by comparing the exact mass values of our metabolomic data with those of the *Aspergillus* natural products database. This analysis revealed 25 known metabolites in *A. nidulans* and 5 in *A. flavus* (Table S3). To determine whether NsdD regulates the production of these metabolites at the transcriptional level, we examined the expression of BGCs in three cell types (Fig. 5) (Table S4). In *A. nidulans* Δ*nsdD*, the production of alternariol, austinol, dehydroaustinol, asterriquinone, and emericellamide C/D was altered, with their backbone genes differentially expressed. In *A. flavus* Δ*nsdD*, *nsdD* deletion affected the biosynthesis of imizoquin D and leporin B, along with the expression of their corresponding genes. These BGCs encode polyketide synthases (*pkgA*, *ausA*, and *easB*), non-ribosomal peptide synthetases (*tdiA*, *easA*, and *imqB*), and TF (*lepB*). Importantly, the relative expression of BGC genes correlated with the abundance of their associated metabolites, with some cases displaying nearly identical trends. These findings suggest that NsdD regulates secondary metabolite production in both *A. nidulans* and *A. flavus* at the transcriptional level.

We also observed a remarkable discrepancy in ST/AF BGC expression during the vegetative stage between the two species (Fig. 5). In *A. nidulans*, most genes within the ST BGC were unaffected by NsdD, except for *stcC* and *stcF*. In contrast, 25 out of 30 genes in the AF BGC were significantly downregulated in *A. flavus* Δ*nsdD*. Notably, the expression level of *aflR*, encoding a key transcriptional regulator of the ST/AF cluster, remained unchanged. Given the established role of NsdD in aflatoxin regulation, these results suggest that NsdD controls ST/AF BGC expression independently of AflR and that proper activation of aflatoxin genes during the vegetative stage is likely essential for successful aflatoxin biosynthesis.

### Identification of potential direct targets of NsdD

To understand the broad regulatory roles of NsdD in *Aspergillus*, we investigated its effect in three different cell types: vegetative cells, asexually developing cells, and conidia. Among these, conidia were selected for further analysis of NsdD direct targets and regulatory networks due to their unicellular, haploid, and isogenic nature (29), which ensures population homogeneity and minimizes experimental variability. Unlike vegetative and asexually developing cells, conidia exist as a synchronized cell population, making them ideal for ChIP-seq experiments and facilitating clearer interpretation of transcriptional regulatory mechanisms.

**Fig 6:**
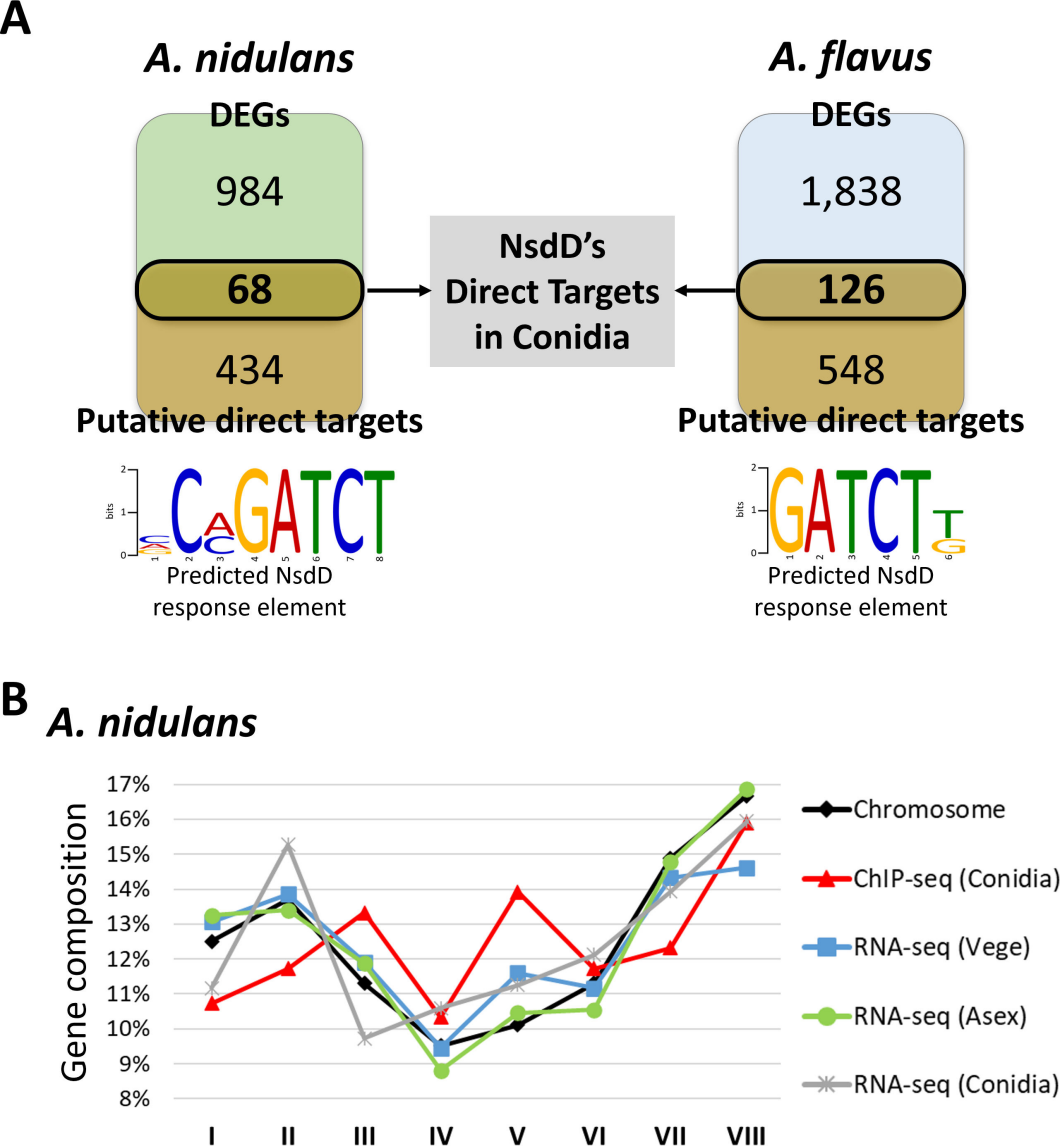
Identification of NsdD direct targets in *A. nidulans* and *A. flavus* conidia. (**A**) Venn diagrams showing the number of putative direct targets of NsdD (ChIP-seq) and differentially expressed genes (DEGs) from RNA-seq analyses in the Δ*nsdD* conidia of *A. nidulans* and *A. flavus*. The overlapping regions indicate genes identified as direct targets of NsdD. Predicted NsdD response elements are shown below each diagram. (**B**) Chromosomal distribution of NsdD-regulated genes in *A. nidulans*. The *y*-axis indicates the percentage of genes from each data set mapped to individual chromosomes. ChIP-seq data sets include direct and putative direct targets, and RNA-seq data sets represent indirect targets of NsdD. Chromosomal information is not yet assigned for *A. flavus* genes.

To identify NsdD direct target genes, we performed ChIP-seq using FLAG-tagged NsdD production strains, TMK13 in *A. nidulans* and THM5 in *A. flavus*. We have identified 502 (4.6% of 10,988 genes) and 674 (5% of 13,485 genes) putative direct target genes whose promoter regions were bound by NsdD in *A. nidulans* and *A. flavus*, respectively. These included key regulators of development and secondary metabolism, such as *veA*, *flbD*, *laeA*, *kapA*, *rosA*, and *steA* in *A. nidulans*, and *veA*, *flbA*, *flbC*, *flbD*, *vosA*, *brlA*, and *rosA* in *A. flavus*. Notably, *nsdD* itself was among the bound genes in both species, suggesting an autoregulatory feedback loop. We next performed motif analysis using Multiple Em for Motif Elicitation (MEME) to identify conserved NsdD binding sites. This analysis revealed a highly conserved NsdD response element (NRE), 5′-GATCT-3′, in both species (Fig. 6A).

Genome-wide regulatory effects of NsdD were assessed by integrating *A. nidulans* ChIP-seq and RNA-seq data. Genes bound by NsdD and/or differentially expressed in Δ*nsdD* were mapped across chromosomes (Fig. 6B). In *A. nidulans*, NsdD-regulated genes were broadly distributed across all chromosomes, indicating a genome-wide regulatory role without any significant chromosomal bias. A similar analysis in *A. flavus* was not possible due to the lack of chromosome-level genome assembly.

To identify genes directly regulated by NsdD, we compared ChIP-seq and RNA-seq data sets, selecting genes both bound by NsdD in their promoter regions and differentially expressed in Δ*nsdD*. This analysis has identified 68 and 126 direct target genes in *A. nidulans* and *A. flavus*, respectively (Fig. 6A) (Table S5). Functional enrichment analyses revealed that NsdD direct targets contribute to species-specific regulatory networks. In *A. nidulans*, these genes were primarily involved in transmembrane transport, whereas in *A. flavus*, they were associated with various biological processes, including transcription (Fig. S4). Notably, although NsdD regulates core developmental and metabolic pathways in both species, its direct targets and downstream regulatory interactions have been rewired, reflecting species-specific adaptations in gene regulatory networks. These findings suggest that NsdD-mediated GRNs have undergone extensive rewiring, with changes in direct target genes and secondary regulatory interactions contributing to distinct transcriptional programs and genome-wide regulatory divergence between *A. nidulans* and *A. flavus*.

### NsdD-mediated GRNs in *Aspergillus*

To elucidate the regulatory mechanisms of NsdD in *Aspergillus*, we constructed gene regulatory networks by integrating ChIP-seq, RNA-seq, and protein–protein interaction (PPI) data sets. In total, we identified 1,486 and 2,512 NsdD-associated genes in *A. nidulans* and *A. flavus* conidia, respectively, by combining ChIP-seq (502 and 674 genes) and RNA-seq (1,052 and 1,964 genes) data sets (Fig. 6A). In comparison, the STRING database predicts only 263 and 262 interactions involving NsdD in *A. nidulans* and *A. flavus*, respectively. These STRING-predicted interactions are largely based on co-mention frequency in published literature and represent putative functional associations rather than experimentally validated regulatory or physical interactions. While useful as a reference, STRING captures only a small portion of NsdD’s regulatory network. In contrast, our experimentally derived data provide the first comprehensive and high-confidence view of NsdD-mediated GRNs in filamentous fungi.

**Fig 7:**
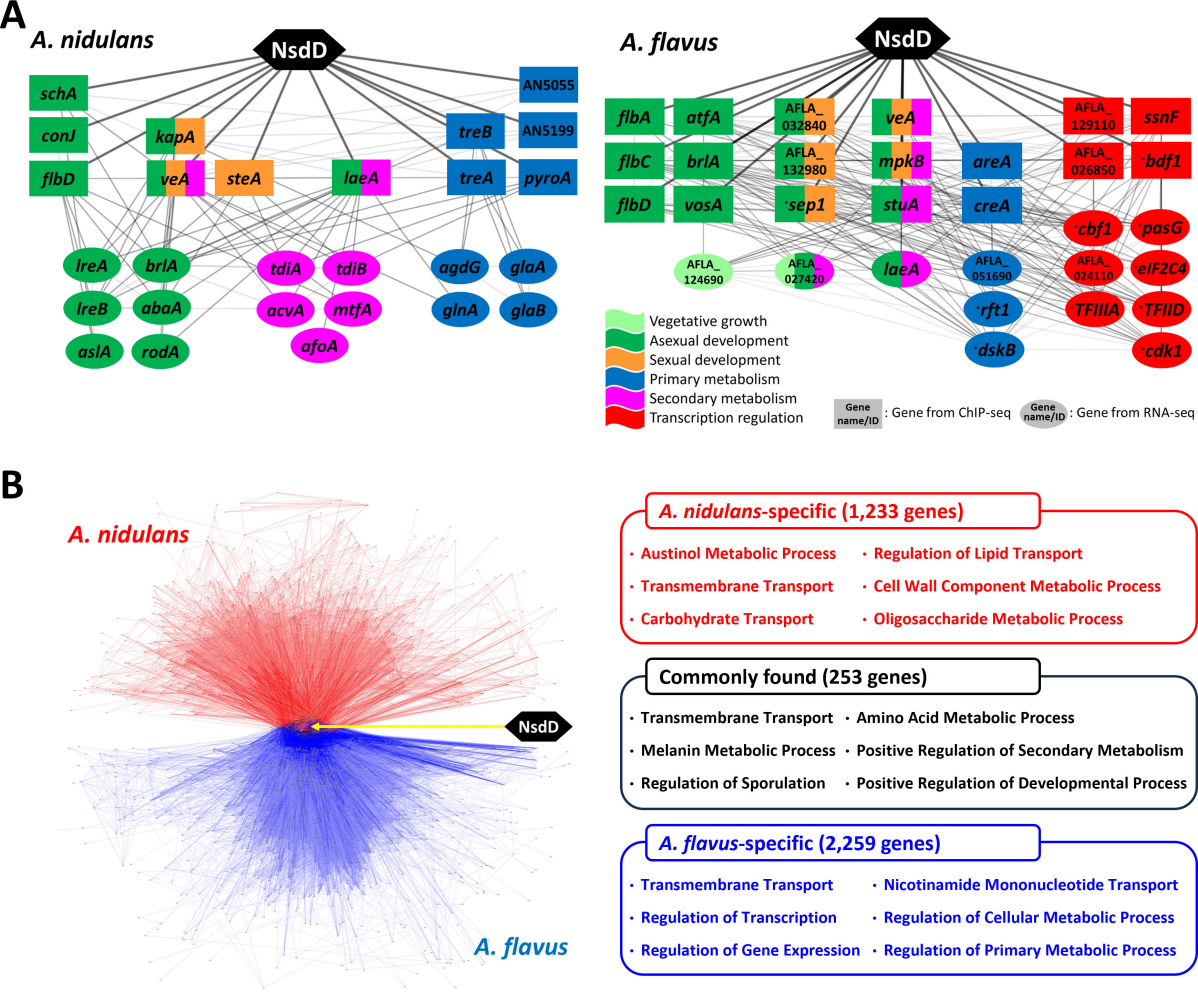
NsdD-mediated GRN analyses in *A. nidulans* and *A. flavus*. (**A**) Core components of NsdD-mediated GRNs in *A. nidulans* and *A. flavus*. Nodes represent genes identified by ChIP-seq (rectangles) or RNA-seq (ellipses), with functional categories color-coded. Edges indicate regulatory or interaction relationships; thicker edges represent direct NsdD binding based on ChIP-seq data. Gene names marked with an asterisk (*) denote predicted functions based on sequence homology. The figure was adapted and modified from reference 33. (**B**) Comparative visualization of NsdD-mediated GRNs. NsdD is centered in the network, with shared target genes (black, 253) placed centrally. *A. nidulans*-specific targets (red; 1,233) extend upward, and *A. flavus*-specific targets (blue; 2,259) extend downward. Representative Gene Ontology terms are shown for each category, with minor modifications for clarity.

Species-specific NsdD-mediated GRNs were constructed in Cytoscape by integrating ChIP-seq, RNA-seq, and PPI data. In the network diagrams, ChIP-seq-derived genes are represented as rectangles and RNA-seq-derived genes as ellipses, and direct NsdD targets are highlighted with thicker edges (Fig. 7A). To identify key components of these networks, we applied the guilt-by-association (GBA) principle (30) to major developmental and metabolic regulators identified in ChIP-seq. Four regulators (*veA*, *flbD*, *laeA*, and *steA*) in *A. nidulans* and seven (*veA*, *flbA*, *flbC*, *flbD*, *vosA*, *brlA*, and *mpkB*) in *A. flavus* were selected (22, 31, 32). Core subnetworks were designed to include approximately 30 genes to balance clarity with informational depth. Genes within these subnetworks were grouped based on predicted functional roles. The *A. nidulans* NsdD core network consisted of 27 genes categorized into four functional groups, while the *A. flavus* network included 31 genes across six categories (Table S6). Homologous sequence information was used to infer potential roles for genes lacking direct functional annotation.

To compare the regulatory roles of NsdD between *A. nidulans* and *A. flavus*, we performed a comparative network analysis and identified 253 orthologous genes that serve as common NsdD targets. In the resulting network visualization (Fig. 7B), these shared targets were positioned centrally, while species-specific targets were placed peripherally: *A*. *nidulans*-specific genes in red and *A. flavus*-specific genes in blue.

To explore the functional roles of shared and species-specific NsdD targets, we performed GO enrichment analysis on the common targets using annotations from *A. nidulans*, which offered more detailed functional categorization than *A. flavus*. Among the 253 common targets, the most enriched GO terms were related to transmembrane transport, primary and secondary metabolism, and developmental regulation. Despite these shared functions, species-specific differences were evident (Fig. 7B). In *A. nidulans*, NsdD-regulated genes were enriched in processes related to cell structure, including phospholipid and α-glucan metabolism, as well as secondary metabolism—particularly austinol biosynthesis. In contrast, *A. flavus*-specific targets were predominantly associated with transcriptional regulation and general cellular metabolism. These findings indicate that while NsdD controls a conserved core set of functions, its broader regulatory network has diverged through changes in direct targets, secondary regulatory relationships, and TF connectivity, resulting in species-specific developmental and metabolic programs.

## DISCUSSION

Sexual and asexual development, along with primary and secondary metabolism, are tightly coordinated processes in *Aspergillus* spp., regulated by multiple TFs and signaling pathways. Among these, NsdD has emerged as a master regulator of fungal development and metabolism. Despite its recognized importance, the genome-wide regulatory mechanisms governed by NsdD have remained largely unexplored. In this study, we employed a network-based multiomics approach to investigate the species-specific functions of NsdD and to uncover the rewiring of NsdD-mediated GRNs in two distantly related species, *A. nidulans* and *A. flavus*.

Our findings reveal that, although NsdD controls conserved biological processes, its direct targets, transcriptional interactions, and downstream regulatory effects differ markedly between species. Transcriptomic analyses demonstrated that NsdD influences gene expression in a cell type-dependent manner, exerting distinct regulatory roles across vegetative hyphae, asexual structures, and conidia. A cross-complementation experiment—introducing *A. nidulans nsdD* into the *A. flavus nsdD* null mutant—further underscored this divergence. While the heterologous expression partially restored WT developmental traits and transcriptomic profiles, it failed to fully replicate native gene expression patterns (Fig. 3). These results suggest that species-specific differences in NsdD conformation, co-regulators, or chromatin accessibility likely contribute to divergent transcriptional outputs.

Metabolomic profiling further highlighted NsdD’s species-specific roles in metabolism. In *A. flavus* ΔnsdD conidia, primary metabolite analysis revealed significant changes in energy-related molecules, including ATP, NADH, and NADPH. Given ATP’s central role in phosphorylation-dependent regulation of the cell cycle, metabolism, growth, and signal transduction, these findings underscore NsdD’s broad influence on fungal physiology (34–36). Similarly, NAD^+^/NADH and NADP^+^/NADP redox couples play critical roles in maintaining the cellular redox state, energy metabolism, gene expression, and signal transduction pathways (37–40). Given the crucial roles of ATP, NAD^+^/NADH, and NADP^+^/NADPH in energy metabolism and gene regulation, along with transcription-related direct targets of NsdD (Fig. 7A), we speculate that in *A. flavus* Δ*nsdD* conidia, elevated energy metabolite levels are strongly linked to widespread transcriptional activity, which in turn demands high energy for chromatin remodeling, RNA synthesis, and protein production.

Beyond primary metabolism, NsdD deletion significantly impacted secondary metabolism, altering the production of several bioactive compounds. Among them, alternariol (AOH), a mycotoxin linked to oesophageal cancer, was markedly downregulated in *A. nidulans* Δ*nsdD* mutants. AOH induces DNA damage via reactive oxygen species generation and topoisomerase activation, leading to strand breaks (41). Previously, AOH biosynthesis in *A. nidulans* was first reported in 2012 (42), and our study confirms that NsdD positively regulates AOH production by controlling *pkgA* expression, as both *pkgA* transcript levels and AOH abundance declined in Δ*nsdD* mutants. Similarly, NsdD deletion led to ARQ overproduction, a class of indolyl benzoquinones with antitumor and antiviral properties (43). ARQs exhibit cytotoxicity against leukemia and potent inhibition of HIV-1 reverse transcriptase (44). In *A. nidulans*, ARQ biosynthesis is controlled by *tdiA-tdiE* genes, which were highly upregulated in the Δ*nsdD* mutants, with *tdiB*, *tdiD*, and *tdiE* increasing up to 16-fold (45). These changes correlated with enhanced ARQ biosynthesis, including terrequinone A, which was absent in WT but present in Δ*nsdD* mutants, suggesting NsdD represses ARQ biosynthesis. In *A. flavus*, leporin B production was dramatically upregulated (~5.7-fold in the Δ*nsdD* mutants). Leporin B induces hexokinase II expression, enhancing glycolysis and lowering blood sugar, making it a promising candidate for type 2 diabetes treatment (46). It also exhibits strong antimicrobial activity against *Candida albicans* and *Staphylococcus aureus* and moderate cytotoxicity against tumor cell lines (MCF7, H460, and SF268) (47). Taken together, these findings suggest that the *nsdD* null mutants could serve as a reservoir for antitumor and antimicrobial secondary metabolites. Further investigation into NsdD-mediated regulation in *Aspergillus* and other Pezizomycotina fungi may reveal its potential for enhancing the production of bioactive compounds with pharmaceutical applications.

To dissect the direct regulatory roles of NsdD, we have performed ChIP-seq analysis in conidia, identifying 502 and 674 putative direct targets in *A. nidulans* and *A. flavus*, respectively. By integrating RNA-seq data, we further refined these numbers, ultimately identifying 68 and 126 genes as direct NsdD targets in each species (33, 48). However, because these analyses were conducted under specific conditions (2-day-old conidia), some putative targets may remain transcriptionally unchanged in this stage but could be regulated in other cell types or growth phases. For instance, in *A. nidulans*, 434 putative direct targets remained unregulated in 2-day-old conidia, whereas 84 and 151 of them were differentially expressed in the Vege and Asex stages, respectively. In line with this, although *brlA* is a known direct target of NsdD in *A. nidulans* (15), it was not detected in our *A. nidulans* ChIP-seq data but appeared in the *A. flavus* data set. This discrepancy may reflect stage- or context-dependent NsdD binding, possibly due to transient genetic interactions in 2-day-old conidia that escape ChIP-seq detection. These findings highlight the complex spatial and temporal regulation of NsdD and suggest that our study captures only a subset of the full NsdD regulatory network, emphasizing the need for further investigation into NsdD-mediated gene regulatory networks across different developmental stages and cellular contexts.

Even though this study has inherent limitations due to condition-specific data, it is the first to elucidate the genome-wide network rewiring of a single TF in *Aspergillus* spp. While the complete NsdD-mediated GRNs have yet to be fully uncovered in each species, our results nonetheless provide valuable genetic insights into both shared and species-specific developmental and metabolic processes. Notably, *A. nidulans* and *A. flavus* Δ*nsdD* mutants exhibited distinct differences in conidium and conidiophore morphology and ST/AF production. A comparison of the core networks offers potential genetic explanations for these divergences. In both species, NsdD regulates most major developmental regulators. However, in *A. flavus*, three genes predicted to encode forkhead box (FOX) proteins and TF, AFLA_032840, AFLA_132980, and AFLA_048110, were uniquely identified. Considering that FhpA/FkhA, a forkhead TF, was recently shown to negatively regulate asexual development in *A. flavus* (49) and FkhB is required for proper conidiophore morphology in *A. nidulans* (50), it is plausible that these FOX proteins contribute specifically to the morphogenesis of asexual structures in *A. flavus*. Regarding ST/AF production, both species share a highly similar BGC structure, including the key transcriptional regulator AflR (Fig. 5) (51). However, *nsdD* deletion resulted in opposite outcomes: in *A. nidulans*, deletion increased ST production, while in *A. flavus*, it almost completely abolished AF production. Our expression analysis revealed that AF BGC was primarily downregulated during the asexual stage in *A. nidulans* Δ*nsdD*, whereas in *A. flavus* Δ*nsdD*, repression was more prominent during the vegetative stage (Fig. S5). Notably, this repression occurred independently of AflR, whose expression level remained unchanged in Δ*nsdD*. These findings suggest that appropriate BGC activation during vegetative growth may be critical for proper AF biosynthesis in *A. flavus*. While further studies are needed to confirm these proposed hypotheses, our network-based approach provides key mechanistic insights into the species-specific regulatory roles of NsdD and its impact on fungal development and secondary metabolism.

In conclusion, this study presents the first genome-wide comparative analysis of NsdD-mediated GRNs in filamentous fungi, illustrating how a single TF can adapt to distinct genetic and environmental contexts through species-specific network rewiring. Although NsdD acts as a global regulator of development and metabolism in both *A. nidulans* and *A. flavus*, its target genes and transcriptional interactions differ markedly, highlighting the evolutionary plasticity of fungal GRNs. Through a network-based multiomics approach, we demonstrate that NsdD coordinates fungal development and metabolism via distinct, species-specific regulatory mechanisms. Future studies focusing on NsdD’s interactions with co-factors, chromatin regulators, and its activity across diverse cell types will be crucial for fully understanding its regulatory complexity. Collectively, our findings provide new insights into the evolution of transcriptional networks in fungi and establish a framework for studying conserved regulators in divergent genomic contexts. Furthermore, given the biomedical significance of several NsdD-regulated secondary metabolites, this work offers valuable perspectives with potential pharmaceutical applications.

## MATERIALS AND METHODS

### *Aspergillus* strains and culture conditions

*Aspergillus* strains used in this study are listed in Table S7. Strains were grown on or in 1% glucose minimal medium (GMM) supplemented as appropriate and incubated at 37°C for *A. nidulans* or 30°C for *A. flavus*. When required, yeast extract was added to GMM at 0.1%–0.5% (11, 52, 53). For conidia quantification, approximately 1 × 10⁵ conidia were spread on solid GMM and incubated for 2 days under standard conditions (37°C for *A. nidulans*, 30°C for *A. flavus*). Conidia were harvested from the entire plate using phosphate-buffered saline and counted with a hemocytometer. These 2-day-old conidia were also used as both inoculation sources and conidial samples. To collect vegetatively growing cells, 5 × 10⁵ conidia/mL were inoculated into 100 mL of liquid GMM and incubated at 220 rpm for 36 h at the respective optimal temperature. For asexual developmental induction, 2 × 10⁶ conidia/mL were inoculated into 100 mL of liquid GMM and cultured for 18 h. The resulting mycelia were transferred to fresh solid GMM, air-exposed, and incubated for an additional 24 h to induce asexual development as previously described (8). For secondary metabolite profiling, 1 × 10⁵ conidia of wild-type and mutant strains were inoculated onto solid GMM and incubated in complete darkness for 14 days at 37°C (*A. nidulans*) or 30°C (*A. flavus*). *Escherichia coli* DH5α was used for plasmid amplification and grown in Luria–Bertani medium supplemented with ampicillin (50 µg/mL, Sigma-Aldrich).

### Generation of *nsdD* complemented strains in *A. flavus*

The oligonucleotides used in this study are listed in Table S8. To complement the Δ*nsdD* mutant, a wild-type (NRRL3357) *nsdD* fragment containing 1.8 kb of the 5′ untranslated region (UTR) and the coding sequence was amplified using primer pair OMK718 and OMK719. The PCR product was digested with *EcoR*I and *Nde*I and cloned into the pHS13 vector (54), which carries a C-terminal 3 × FLAG tag and the *trpC* terminator. The resulting plasmid (pHM1) was validated by PCR and restriction digestion, and its amino acid sequence was confirmed by genomic sequencing using primers OHM40, OHM42, and OHM39. pHM1 was transformed into the Δ*nsdD* mutant strain LNJ11. At least three independent transformants (designated THM5) expressing wild-type NsdD with a C-terminal 3 × FLAG tag under the control of its native promoter were isolated and verified. To generate a cross-complemented strain expressing *A. nidulans nsdD* in *A. flavus*, the *A. nidulans* wild-type (RJMP1.59) *nsdD* open reading frame was amplified and fused to the 1.8 kb 5′ UTR of *A. flavus nsdD*, then cloned into the pHS13 vector. The resulting plasmid (pHM2) was confirmed by PCR, restriction digestion, and sequencing. pHM2 was transformed into the Δ*nsdD* mutant strain TNJ108, and at least three independent cross-complemented transformants (designated THM6) were isolated and validated.

### Nucleic acid isolation and manipulation

Genomic DNA was isolated as previously described (55). Briefly, a loopful of conidia (approximately 10³ to 10⁴ per loop) from solid culture was inoculated into 10 mL of liquid GMM in a sterile petri dish and incubated for 12–15 h at 37°C (*A. nidulans*) or 30°C (*A. flavus*). The resulting semitransparent mycelial mat was collected, gently squeeze-dried to remove excess moisture, and then freeze-dried. The dried fungal tissue was ground into a fine powder using a motor-spatula tool, and high-quality genomic DNA was subsequently extracted. Total RNA isolation and Northern blot analysis were performed as previously described (8, 56).

### Protein extraction and Western blot analysis

Western blot analysis of NsdD was performed as previously described (57). Briefly, 2-day-old conidia (2 × 10⁸) of the THM5 strain were harvested and resuspended in spore lysis buffer supplemented with a 1 × protease inhibitor cocktail. Cells were homogenized using a mini-beadbeater with 0.5 mm zirconia/silica beads. Protein concentrations were determined using the Bio-Rad Protein Assay (Bio-Rad). Approximately 15 µg of total proteins per sample was separated by SDS-PAGE on a 4%–15% gradient gel (Bio-Rad) and transferred to an Immobilon-P PVDF membrane (Millipore). The membrane was blocked with blocking buffer, incubated with monoclonal anti-FLAG antibody (clone M2, Sigma-Aldrich), and subsequently incubated with horseradish peroxidase-conjugated antimouse IgG (Millipore). The membrane was developed using Amersham enhanced chemiluminescence detection reagents (GE Healthcare).

### Primary metabolite analysis

Sample preparation for primary metabolite analysis was performed as previously described (48). Briefly, 2-day-old conidia (2 × 10⁸) of each strain were resuspended in 500 µL of high-performance liquid chromatography (HPLC)-grade acetonitrile:methanol:water (40:40:20, vol/vol/vol) with 300 µL of 0.5 mm zirconia/silica beads and homogenized using a mini-beadbeater. The homogenized samples were centrifuged, and the supernatants were filtered through 0.45 µm PTFE Mini-UniPrep filters (Agilent) and used for metabolite analysis.

Samples were analyzed using a previously described method (58, 59). The HPLC-MS consisting of a Dionex ultra-high-performance liquid chromatography (UHPLC) instrument coupled by electrospray ionization (negative mode) to a hybrid quadrupole-high-resolution mass spectrometer (Q Exactive orbitrap, Thermo Fisher Scientific) operating in full-scan mode was performed. Differentially accumulated metabolites between wild-type and Δ*nsdD* strains were identified based on the following criteria: *P* value of <0.05, peak intensity of >10⁶, and absolute log_2_ fold change of >1. Known metabolites were identified by comparing exact mass values (with an accuracy of ±0.001 Da) and retention times (±1.5 range) to those of authentic standards (Sigma-Aldrich).

### Secondary metabolite analysis

For secondary metabolite profiling, wild-type and Δ*nsdD* strains were point-inoculated onto solid GMM, sealed with Parafilm (Bemis), and incubated at 37°C (*A. nidulans*) or 30°C (*A. flavus*) for 14 days. Entire agar plates were extracted by blending with 300 mL of methanol, followed by sonication for 60 min, and left at room temperature for 24 h. The extracts were filtered under vacuum to remove solid residue, and the organic phase was separated and dried over anhydrous magnesium sulfate. After additional filtration, the solvent was evaporated to obtain dried organic extracts. Each extract was resuspended in acetonitrile to a final concentration of 10 mg/mL and filtered through a 0.45 µm Acrodisc syringe filter with a nylon membrane (Pall Corporation). Samples were analyzed by UHPLC-HRMS in both electrospray ionization (ESI)-positive and ESI-negative modes within the *m*/*z* range from 150 to 1,500. UHPLC-HRMS data were processed using a Thermo Scientific Vanquish UHPLC system (Waltham) connected to a Q Exactive Orbitrap mass spectrometer (Thermo Fisher Scientific). A Zorbax Eclipse XDB-C18 column (2.1 × 150.0 mm, 1.8 µm) was used at a flow rate of 0.2 mL/min. Water with 0.05% formic acid and acetonitrile (MeCN) with 0.05% formic acid were used with the following gradient: 0 min, 80% aq. MeCN; 15 min, 2% aq. MeCN; 20 min, 2% aq. MeCN.

Raw data were processed using XCMS online (v.3.7.1, Scripps Research Institute). Differentially produced metabolites were identified using the same criteria applied for primary metabolites, excluding exact matching with chemical standards. Known secondary metabolites were identified by matching exact mass values (within three decimal digits of accuracy) to annotated compounds in the NPAtlas microbial natural products database (v.2021_08) (60) for *A. nidulans* and *A. flavus*.

### RNA sequencing analysis

Total RNA was extracted from each sample and submitted to Novogene (Beijing, China) for quality assessment, library construction, and mRNA sequencing. RNA quality was verified using 1% agarose gel electrophoresis, a Qubit (v.3.0) fluorometer (Thermo Fisher), and an Agilent 2100 Bioanalyzer. Only samples with RNA concentrations of ≥20 ng/µL, OD260/280 ratios of >2.0, and RNA integrity numbers of ≥6.3 were used for library preparation. Strand-specific RNA-seq libraries were constructed using the Illumina TruSeq strand-specific RNA library preparation system. Libraries with insert sizes of 250–300 bp were sequenced on the Illumina NovaSeq 6000 platform with a 150 bp paired-end read strategy. On average, more than 3.9 × 10⁷ high-quality reads and 5.7 × 10⁹ clean bases were obtained per sample, with an average base error rate below 0.03%.

Reference genomes and annotation files were obtained from NCBI: *A. nidulans* (GCF_000149205.2) and *A. flavus* (GCF_000006275.2). Clean reads from vegetative (Vege) and asexual (Asex) samples were mapped to the respective genomes using TopHat2 (v.2.0.12) (61), while reads from conidia samples were mapped using HISAT2 (v.2.1.0) (62), all with default parameters unless otherwise specified. More than 95% of conidia reads and over 84% of Vege and Asex reads were successfully mapped to the genome. Read counts were generated using HTSeq (v.0.6.1) (63) for Vege and Asex samples and FeatureCounts (v.1.5.0) (64) for conidia samples. Gene expression levels were quantified as fragments per kilobase of transcript per million mapped reads (FPKM) with 100% coverage across samples. For differential expression analysis, FPKM values were quantile-normalized using the edgeR package in R (v.4.1.2). Genes were classified as differentially expressed if they met both statistical and biological thresholds: a *P* value of <0.05 (two-tailed Student’s *t*-test) and a greater than twofold change in raw FPKM values. Fold changes were calculated as the log_2_ ratio of WT to Δ*nsdD* FPKM values. A negative log_2_ fold change indicates upregulation in Δ*nsdD* (i.e., NsdD represses the gene in WT), while a positive value indicates downregulation (i.e., NsdD activates the gene in WT).

### Functional enrichment analysis

GO enrichment analysis was performed using the FungiDB platform (65). Analyses were conducted using the “Biological Process” ontology without restricting to GO Slim terms. Enriched GO terms with *P* values of <0.05 were considered statistically significant. To prioritize biological relevance, enriched terms were sorted by ascending *P* values and descending numbers of associated genes. Final selection of the most representative functional categories was based on both statistical significance and gene set size.

### Chromatin immunoprecipitation sequencing analysis

ChIP-seq was performed as previously described (15, 66, 67) with some modifications. Two-day-old conidia (2 × 10⁹) of TMK13 (*A. nidulans*) and THM5 (*A. flavus*) were cross-linked with 1% formaldehyde, resuspended in spore lysis buffer, and homogenized using a mini-beadbeater with 0.5 mm zirconia/silica beads. Lysates were sonicated for five to seven cycles (60 s on, 60 s off) with a sonifier (57), centrifuged, and diluted in ChIP dilution buffer. ChIP was performed using the MAGnify ChIP System (Invitrogen) with 1 µg of monoclonal anti-FLAG antibody (Sigma-Aldrich). Antirabbit IgG was used as a negative control, and input DNA served as a positive control. Enriched DNA was recovered for ChIP-PCR and ChIP-seq. Primer sequences used for ChIP-PCR are listed in Table S8.

ChIP DNA from *A. nidulans* was sequenced by ProteinCT (Madison, WI), and *A. flavus* samples were processed by Novogene. Libraries were prepared using the TruSeq ChIP Library Prep Kit (Illumina) and sequenced on the Illumina HiSeq2500 (*A. nidulans*) or NovaSeq 6000 (*A. flavus*) platform. Each sample yielded >8 million (*A. nidulans*) or >18 million (*A. flavus*) reads with DNA fragment sizes ranging from 50 to 150 bp. Reads were mapped to reference genomes using Bowtie2 (*A. nidulans*) (68) or BWA (v.0.7.12) (*A. flavus*) (69). Peaks were called using HOMER (*A. nidulans*) (70) or MACS2 (v.2.1.0) (*A. flavus*) (71). Identification of direct NsdD targets was performed as described in reference 33, defining them as genes with ChIP-seq peaks located within 1.5 kb upstream of the translation start site. The NRE motif was identified using MEME-ChIP (v.4.12.0) (72, 73), with default parameters except for the number of motifs (10) and motif length (5–21 bp).

### Gene regulatory network analysis

Species-specific NsdD-mediated GRNs were defined as PPI networks consisting of NsdD, its putative direct targets identified by ChIP-seq, and DEGs identified through transcriptomic analysis of conidia samples. Known PPIs for *A. nidulans* and *A. flavus* were retrieved from the STRING database (v.11.5) (74) by matching protein and gene identifiers using the “protein.aliases” reference table. Edges representing functional associations between proteins, as defined by STRING PPI data with a confidence score of ≥150, were initially selected. From this set, only interactions in which both nodes corresponded to NsdD direct targets and/or DEGs were retained, forming the backbone of each species-specific network.

For network visualization, NsdD was placed at the center, with ChIP-seq-derived genes arranged in an inner circle and RNA-seq-derived DEGs positioned in the outer layer. To identify core regulatory modules, we applied the GBA principle, extending the network to include first neighbors of the backbone components. These expanded networks were used to extract the core sections of species-specific GRNs. In these core sections, we highlighted interactions between NsdD and key transcriptional regulators identified by ChIP-seq, while DEGs from RNA-seq were placed in the lower region of the diagrams. Rectangles and ellipses were used to denote genes identified from ChIP-seq and RNA-seq, respectively, and were color-coded by predicted functional category: vegetative growth (pale green), asexual development (green), sexual development (deep saffron), primary metabolism (blue), secondary metabolism (magenta), and transcription regulation (red). All network visualizations were generated using Cytoscape (v.3.9.1) (75).

For comparative network analysis, orthologous genes between *A. nidulans* and *A. flavus*, including *nsdD*, were identified based on a previously established ortholog list (73). Orthologs shared between the two species-specific networks were manually positioned near NsdD at the network center. Species-specific genes were visualized following the same layout strategy as in individual network constructions.

## Data Availability

As gene annotation information for *Aspergillus flavus* (AFLA_ ID format) is no longer publicly available online, the corresponding annotation file is provided as supplemental material. All RNA-seq and ChIP-seq data that are relevant to the publication are freely available from the NCBI Gene Expression Omnibus database under accession number GSE219142 (*Aspergillus nidulans* RNA-seq and ChIP-seq, accession numbers GSE219140 and GSE219139, respectively; *A. flavus* RNA-seq and ChIP-seq, accession numbers GSE219138 and GSE219137, respectively). The GRN files are available at https://drive.google.com/drive/folders/1k5j9U_DXGb8O8e-3ftXQr1zqzd4UD23W?usp=share_link.

## SUPPLEMENTAL MATERIAL

Supplemental figures mbio.01181-25-s0001.docx Figures S1 to S5.

Gene annotation mbio.01181-25-s0002.xlsx The old version of *Aspergillus flavus* gene annotation (AFLA_ ID format).

Supplemental tables mbio.01181-25-s0003.xlsx Tables S1 to S8.

ASM does not own the copyrights to Supplemental Material that may be linked to, or accessed through, an article. The authors have granted ASM a non-exclusive, world-wide license to publish the Supplemental Material files. Please contact the corresponding author directly for reuse.
