# Supplementary material for: Species-specific gene regulatory network rewiring mediated by the GATA-type regulator NsdD in Aspergillus
Source: mBio. 2025 Jul 3;16(8):e01181-25. doi: 10.1128/mbio.01181-25 (PMC12345274; doi:10.1128/mbio.01181-25)
Supplement: Supplemental figures — Figures S1 to S5. [file mbio.01181-25-s0001.docx]

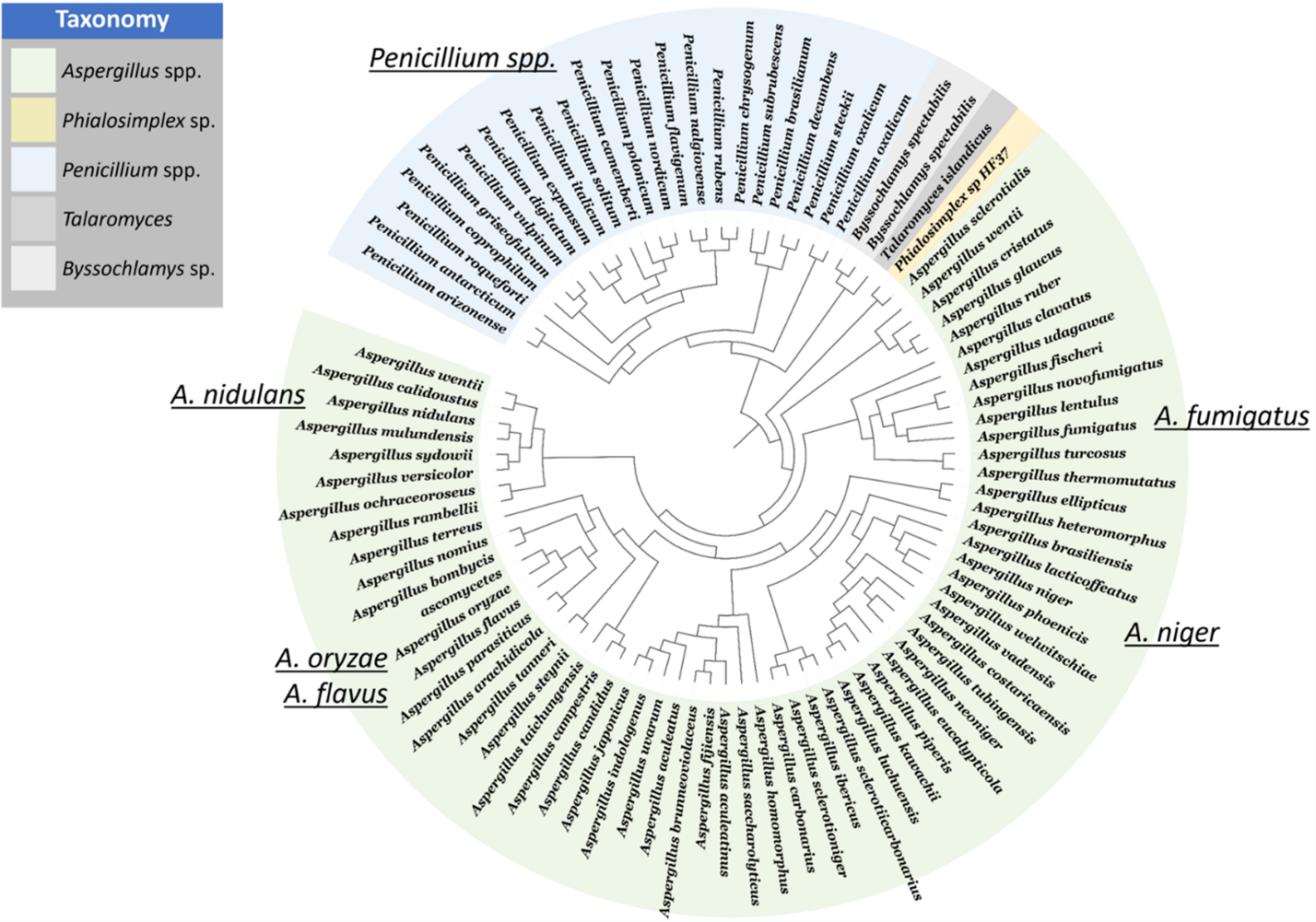


**Figure S1. Phylogenetic tree of NsdD proteins in *Aspergillus* species and related genera.** The maximum-likelihood phylogenetic tree was constructed based on the amino acid sequences of NsdD orthologs identified in *Aspergillus* species and closely related genera including *Penicillium*, *Phialosimplex*, *Talaromyces*, and *Byssochlamys*. Taxonomic groups are color-coded. Selected clades are labeled to highlight major *Aspergillus* sections including *A. nidulans*, *A. fumigatus*, *A. niger*, *A. oryzae*, and *A. flavus*.


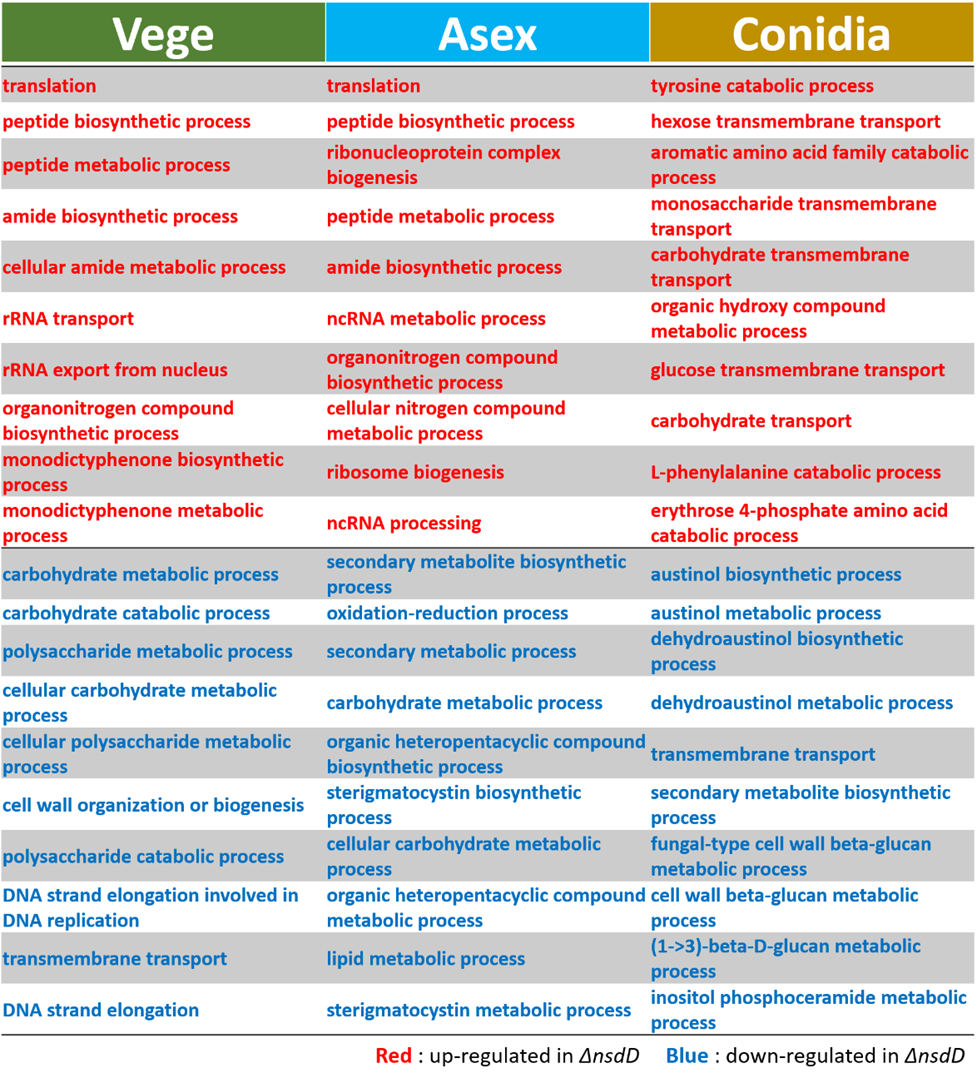


**Figure S2. Gene Ontology (GO) enrichment analysis of differentially expressed genes (DEGs) in *A. nidulans* Δ*nsdD*.** GO terms enriched in the Vege, Asex, and Conidia stages of the *nsdD* deletion mutant are shown. Biological processes associated with genes up-regulated in Δ*nsdD* are shown in red, whereas those associated with down-regulated genes are shown in blue.


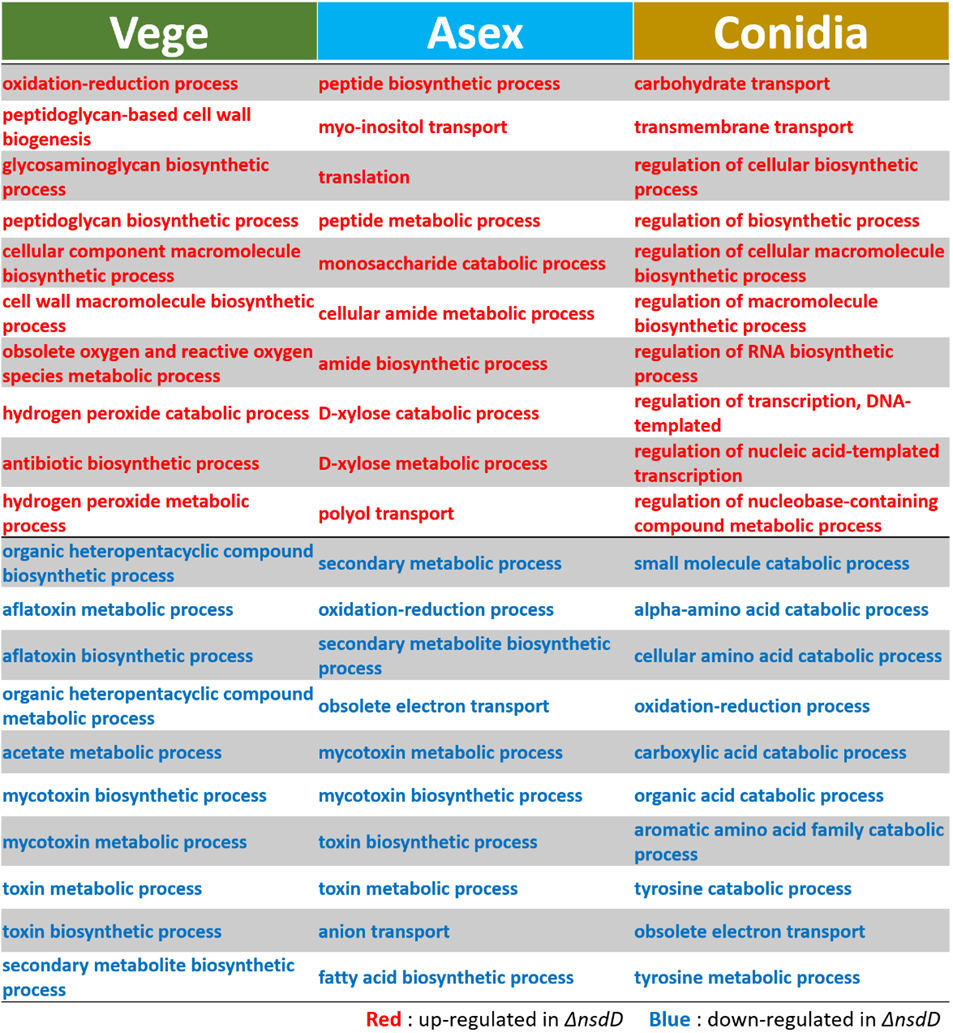


**Figure S3. Gene Ontology (GO) enrichment analysis of differentially expressed genes (DEGs) in *A. flavus* Δ*nsdD*.** GO terms enriched in the Vege, Asex, and Conidia stages of the *nsdD* deletion mutant are shown. Biological processes associated with genes up-regulated in Δ*nsdD* are shown in red, whereas those associated with down-regulated genes are shown in blue.


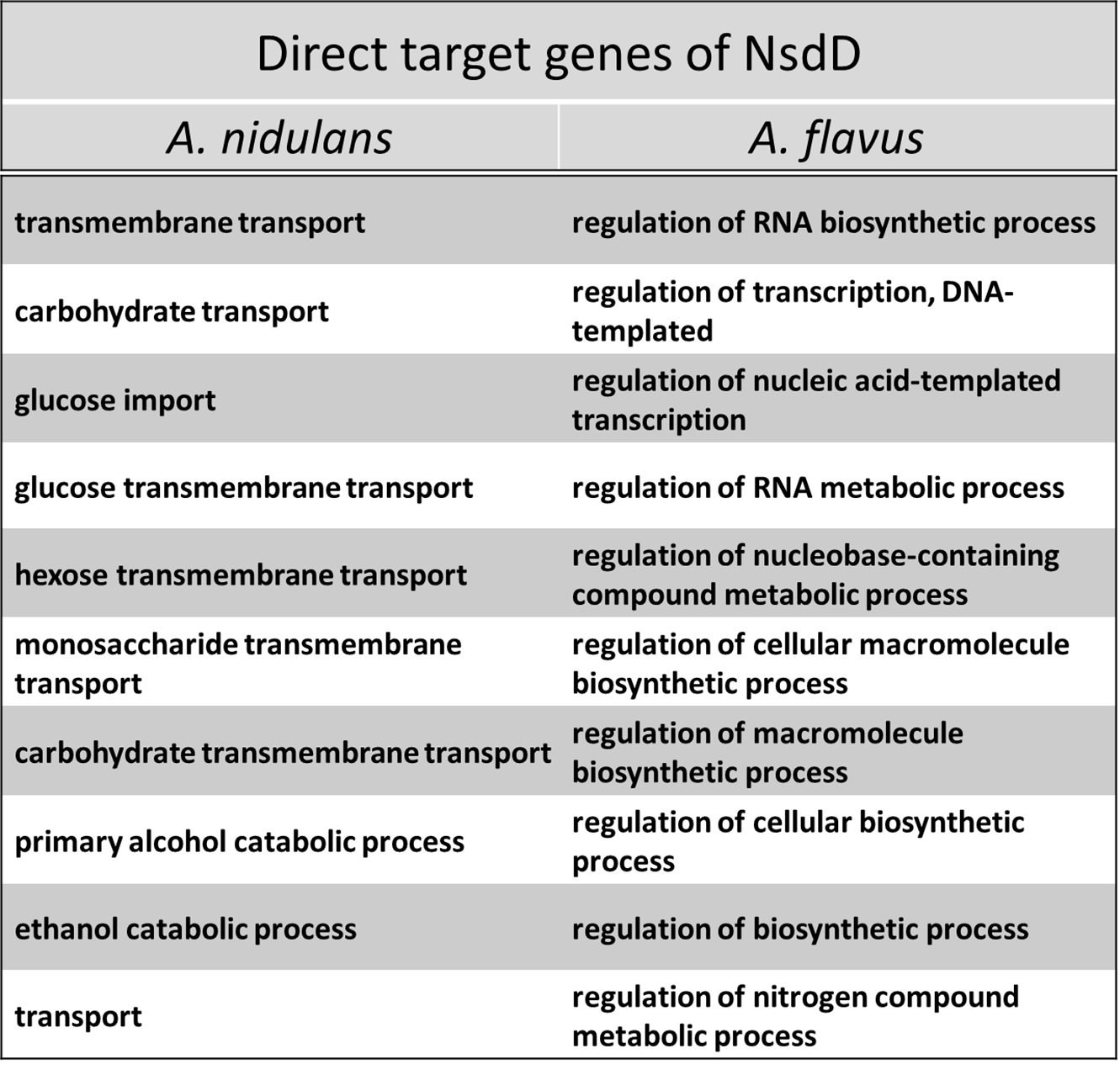


**Figure S4. Gene Ontology (GO) enrichment analysis of direct target genes of NsdD in *A. nidulans* and *A. flavus*.** Enriched biological processes reflect conserved and species-specific regulatory roles of NsdD in fungal development and metabolism.


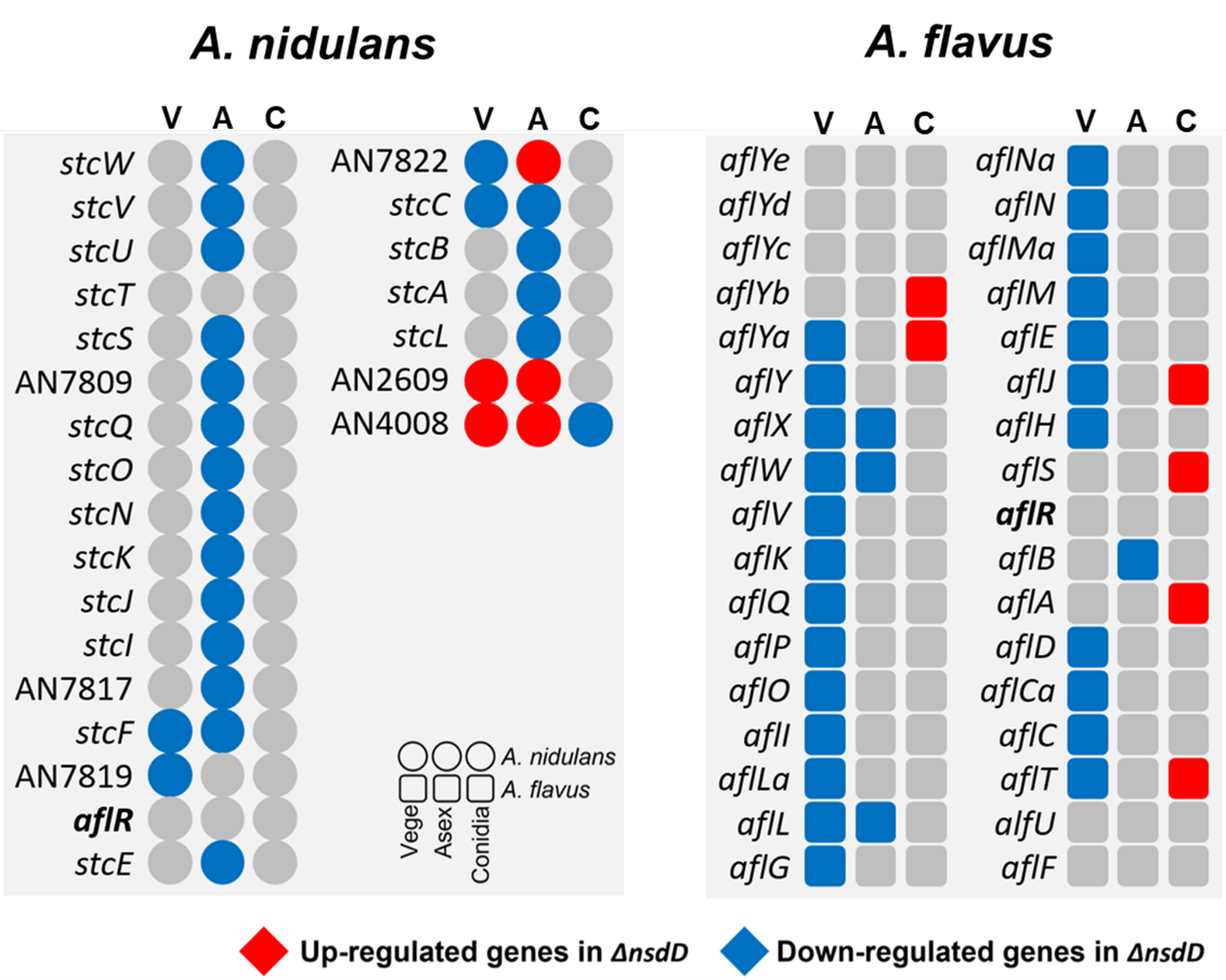


**Figure S5. Expression profiles of sterigmatocystin (ST) and aflatoxin (AF) biosynthetic gene clusters in *A. nidulans* and *A. flavus*.** The heatmap shows the transcriptional changes of ST/AF genes across three developmental stages—Vege, Asex, and Conidia—in Δ*nsdD* mutants compared to WT. Circles represent *A. nidulans* genes, and squares represent *A. flavus* genes. Red indicates genes up-regulated in Δ*nsdD*, while blue indicates down-regulation. Genes without significant expression changes are shown in gray.
